# The Ramazzini Institute 13-week pilot study on glyphosate and Roundup administered at human-equivalent dose to Sprague Dawley rats: effects on the microbiome

**DOI:** 10.1186/s12940-018-0394-x

**Published:** 2018-05-29

**Authors:** Qixing Mao, Fabiana Manservisi, Simona Panzacchi, Daniele Mandrioli, Ilaria Menghetti, Andrea Vornoli, Luciano Bua, Laura Falcioni, Corina Lesseur, Jia Chen, Fiorella Belpoggi, Jianzhong Hu

**Affiliations:** 1 Department of Genetics and Genomic Sciences, Icahn School of Medicine at Mount Sinai, 1428 Madison, New York, NY 10029 USA; 2 Department of Thoracic Surgery, Jiangsu Key Laboratory of Molecular and Translational Cancer Research, Cancer Institute of Jiangsu Province, Nanjing Medical University Affiliated Cancer Hospital, Nanjing, China; 3 Cesare Maltoni Cancer Research Center (CMCRC), Ramazzini Institute (RI), Via Saliceto, 3, 40010 Bentivoglio, Bologna, Italy; 4 Department of Veterinary Medical Sciences, University of Bologna, Bologna, Italy; 5 Department of Agricultural Sciences, University of Bologna, Bologna, Italy; 6 Department of Environmental Medicine and Public Health, Icahn School of Medicine at Mount Sinai, 1428 Madison, New York, NY 10029 USA

**Keywords:** Roundup, Glyphosate, Gut microbiome, Early developmental stage

## Abstract

**Background:**

Glyphosate-based herbicides (GBHs) are broad-spectrum herbicides that act on the shikimate pathway in bacteria, fungi, and plants. The possible effects of GBHs on human health are the subject of an intense public debate for both its potential carcinogenic and non-carcinogenic effects, including its effects on microbiome. The present pilot study examines whether exposure to GBHs at doses of glyphosate considered to be “safe” (the US Acceptable Daily Intake - ADI - of 1.75 mg/kg bw/day), starting from in utero, may modify the composition of gut microbiome in Sprague Dawley (SD) rats.

**Methods:**

Glyphosate alone and Roundup, a commercial brand of GBHs, were administered in drinking water at doses comparable to the US glyphosate ADI (1.75 mg/kg bw/day) to F0 dams starting from the gestational day (GD) 6 up to postnatal day (PND) 125. Animal feces were collected at multiple time points from both F0 dams and F1 pups. The gut microbiota of 433 fecal samples were profiled at V3-V4 region of 16S ribosomal RNA gene and further taxonomically assigned and assessed for diversity analysis. We tested the effect of exposure on overall microbiome diversity using PERMANOVA and on individual taxa by LEfSe analysis.

**Results:**

Microbiome profiling revealed that low-dose exposure to Roundup and glyphosate resulted in significant and distinctive changes in overall bacterial composition in F1 pups only. Specifically, at PND31, corresponding to pre-pubertal age in humans, relative abundance for *Bacteriodetes* (*Prevotella*) was increased while the *Firmicutes (Lactobacillus)* was reduced in both Roundup and glyphosate exposed F1 pups compared to controls.

**Conclusions:**

This study provides initial evidence that exposures to commonly used GBHs, at doses considered safe, are capable of modifying the gut microbiota in early development, particularly before the onset of puberty. These findings warrant future studies on potential health effects of GBHs in early development such as childhood.

**Electronic supplementary material:**

The online version of this article (10.1186/s12940-018-0394-x) contains supplementary material, which is available to authorized users.

## Background

Glyphosate (IUPAC chemical name N-(phosphonomethyl) glycine) is the active ingredient of the most widely applied herbicide worldwide, glyphosate-based herbicides (GBHs), including the best-known formulation Roundup. The substance glyphosate was initially discovered in 1950 by a Swiss chemist, Henri Martin, at the pharmaceutical company Cilag [1]. Its herbicidal properties were not discovered for another 20 years. Since glyphosate was patented in 1974 by Monsanto as a herbicide, approximately 9.4 million tons of GBHs have been sprayed, nearly half a pound of glyphosate on every cultivated acre of land globally [2]. Furthermore, after the introduction of genetically modified (GM) crops that are glyphosate-tolerant in 1996, usage of GBHs has skyrocketed; about two-thirds of the total GBHs usage took place in recent decades. According to the National Academy of Sciences report [3], in 2014 alone, annual glyphosate usage in agriculture industry exceeded 110 million kilograms. Besides GM crops, farmers also apply GBHs on non-GM crops in order to accelerate the harvest. This practice, also known as desiccation, has led to significant dietary exposure to the residues of glyphosate and its primary metabolite AMPA (aminomethylphosphonic acid) [4, 5].

The primary herbicidal function of glyphosate is to inhibit a key plant enzyme, namely 5-enolpyruvylshikimate-3-phosphate synthase (EPSPS). This enzyme participates in the biosynthesis of aromatic amino acids (phenylalanine, tyrosine and tryptophan) via the shikimate pathway in bacteria, fungi, and plants. The only enzyme known to catalyze a similar reaction in bacteria is the enzyme MurA (UDP-N-acetylglucosamine enolpyruvyl transferase, EC 2.5.1.7), which catalyzes the first committed step in the synthesis of the peptidoglycan layer of the bacterial cell. Growth and survival of bacteria relies on the functionality of the enzyme MurA that is the target of the broad-spectrum antibiotic fosfomycin. Glyphosate appears to occupy a binding site of MurA, mimicking an intermediate state of the ternary enzyme-substrates complex [6]. The similarity between the two enolpyruvyl transferases EPSPSe and MurA appears to clarify the antibacterial activity of Glyphosate. As the EPSPS-driven pathway does not exist in vertebrate cells, many scientists and environmental regulating agencies believed that glyphosate would impose minimal risks to mammals, in particular, humans [7–9]. For this reason, the shikimate pathway has been the target for the development of new anti-microbial and anti-parasite agents. In fact, glyphosate formulation has been patented as anti-parasite drug [10]. However, several emerging evidence suggested that glyphosate or GBHs (such as Roundup) can adversely affect mammalian biology via multiple mechanisms [11–13]. Downstream analyses of the functional interactions between the host and its microbiome are starting to provide mechanistic insights into these interactions. The mechanisms in which the enteric microbiome modulates specific effects on the host is not completely clear, although several mediators have been suggested as potential vehicles for such influence and might behave as effectors, enzyme cofactors and signal molecules. Such mediators include lipopolysaccharides, peptidoglycans, short-chain fatty acids, neurotransmitters and gaseous molecules [14, 15]. Recent advances in characterizing the composition and function of individual microbial species and complex microbial communities are revealing the importance of microbial metabolism for the host immune system [16]. The gut microbiota produces an extremely diverse metabolite repertoire (such as propionic acid, a short-chain fatty acids) from the anaerobic fermentation of exogenous undigested dietary components (such as fibers) that reach the colon, as well as endogenous compounds that are generated by microorganisms and the host [17]. The single layer of epithelial cells that makes up the mucosal interface between the host and microorganisms allows microbial metabolic products to gain access to and interact with host cells, and thus influence immune responses and disease risk, in particular at high concentration [18].

GBHs have been reported to alter microbiota in soil [19], plants [20] and animals [21, 22]. A number of studies have suggested that GBHs could act as antibiotics in the mammalian gut microbiome. Recent studies have raised concerns about the health effects of glyphosate on gut microbiota of farm animal when fed feed containing residues of glyphosate. For example, farm animal studies linked epidemics of *C. Botulinum*-mediated diseases in dairy cows [23] to glyphosate exposure. It has been proposed that glyphosate has a potential inhibiting effect on growth of commensal bacteria, normally occupying the gut of farm animals. For example, a reduction of such beneficial bacteria could be a predisposing factor for Campylobacteriosis (*campylobacter* infection) described as an emerging food-borne disease [24]. Poultry is a major reservoir and source of transmission of campylobacteriosis to humans [22]. Furthermore, GBHs were also found to be capable of inducing multiple-antibiotic resistance phenotype in potential pathogens [25]. Therefore, GBHs may have the potential to modify the animal and human microbiota, which, in turn, could influence human health. However, up to date, no direct evidence has been reported to suggest any interplay between GBHs exposure and the microbiome in humans, especially during early development or in animal models exposed to GBH with low dosage relevant to humans. As denoted in the Developmental Origins of Health and Disease (DOHaD) paradigm [26], early environmental exposures are important to human health. In particular, the prenatal and neonatal period represent a narrow but critical window of susceptibility to myriad environmental exposures and conditions with potentially lifelong impacts on health and disease. A number of human and animal studies [27–29] associate several diseases with early-life imbalances of the gut microbiota, but it was recently pointed out the need for further evidence that GBHs, in particular at environmentally relevant doses, can result in disturbances in the gut microbiome of human and animal populations with negative health implications [30]. Furthermore, exploring the effects of GBHs on the microbiota from early-life until adulthood in different windows of susceptibility, may give a more accurate portrayal of the microbial conditions that are involved in pathogenesis. Possible alterations of the mammalian gut microbiota and its metabolites by environmental concentrations of GBHs in early development, starting from in utero, have never been explored in a controlled laboratory animal study. The present pilot study examines whether exposure to GBHs at doses of glyphosate considered to be “safe”, the US ADI of 1.75 mg/kg bw/day, defined as the chronic Reference Dose (cRfD) determined by the US EPA [31], affect the composition and diversity of the gut microbiome at early developmental stages in Sprague-Dawley rats.

## Methods

### Experimental model

The entire animal experiment was performed following the rules by the Italian law regulating the use and treatment of animals for scientific purposes (Legislative Decree No. 26, 2014. Implementation of the directive n. 2010/63/EU on the protection of animals used for scientific purposes. - G.U. General Series, n. 61 of March 14th 2014). All animal study procedures were performed at the Cesare Maltoni Cancer Research Centre/Ramazzini Institute (CMCRC/RI) (Bentivoglio, Italy). The study protocol was approved by the Ethical Committee of Ramazzini Institute. The protocol of the experiment was also approved and formally authorized by the ad hoc commission of the Italian Ministry of Health (ministerial approval n. 710/2015-PR). The CMCRC/RI animal breeding facility was the supplier for the Sprague-Dawley (SD) rats. Female breeders SD rats were placed individually in Polycarbonate cage (42x26x18cm; Tecniplast Buguggiate, Varese, Italy) with a single unrelated male until evidence of copulation was observed. After mating, matched females were housed separately during gestation and delivery. Newborns were housed with their mothers until weaning. Weaned offspring were co-housed, by sex and treatment group, not more than 3 per each cage. Cages were identified by a card indicating: study protocol code, experimental and pedigree numbers, dosage group. A shallow layer of white fir wood shavings served as bedding (supplier: Giuseppe Bordignon, Treviso, Italy). Analysis of chemical characteristics (pH, ashes, dry weight, specific weight) and possible contamination (metals, aflatoxin, polychlorobiphenyls, organophosphorus and organochlorine pesticides) of the bedding was performed by CONSULAB Laboratories (Treviso, Italy). The cages were placed on racks, inside a single room prepared for the experiment at 22 °C ± 3 °C temperature and 50 ± 20% relative humidity. Daily checks on temperature and humidity were performed. The light was artificial and a light/dark cycle of 12 h was maintained. Husbandry factors stress-related were controlled: rats were kept together (same room, same rack, no more than 3 per cage) and we did not relocate cages. Noise and handling time were minimized [32].

### Experimental protocol

Two groups of SD rat dams and relative pups were treated with either glyphosate or Roundup diluted in drinking water at the glyphosate concentration of 1.75 mg/kg bw/day. There were in total 24 F0 dams, entire litter at postnatal day (PND) 7 and PND 14, 108 F1 offspring at PND 31 and PND 57 and 60 F1 at PND 125 in this study. The F0 female breeders received the treatment through drinking water from gestation day (GD) 6 to the end of lactation. During pregnancy and lactation, embryos and offspring (F1) were all retained in the litter and might receive the test compounds mainly through their dams (F0). After weaning on PND 28 offspring were randomly distributed in two cohorts: animals belonging to the 6-week cohort were sacrificed at PND 73 ± 2, i.e. 6 weeks after weaning, animals belonging to the 13-week cohort were sacrificed at PND 125 ± 2, i.e. 13 weeks after weaning. The F1 offspring might receive the treatment from their dams starting from in utero and mainly through milk during lactation. After weaning, the offspring (F1) were treated through drinking water until sacrifice.

**Fig. 1:**
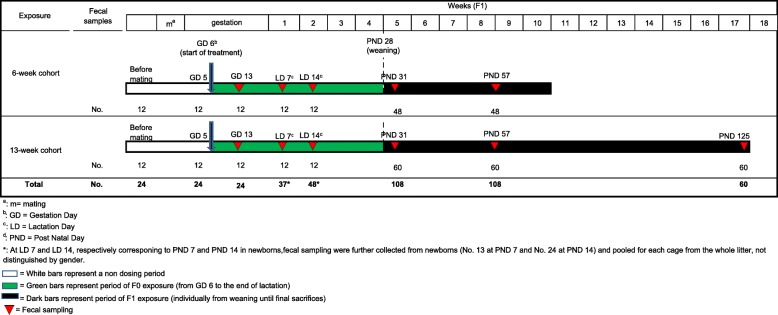
Timeline of the experimental animal treatment and fecal sample collection

The timeline of the experimental animal treatment and fecal sample collection is shown in Fig. 1. As illustrated, rat fecal samples were individually collected from all animals of the F0 generation (8 dams) from each group before mating, at GD 5 (before the starting of the treatment), GD 13, lactation day (LD) 7 and LD 14. Fecal samples were also collected from 108 F1 pups, 18 males and 18 females from each group during lactation at PND 7 and PND 14 (corresponding to LD 7 and 14 for dams), before the achievement of puberty at PND 31, after puberty at PND 57 and in adulthood at PND 125. Due to technical difficulty to identify fecal samples from individual pups during lactation, only pooled samples at PND 7 and PND 14 were collected for each cage from the whole litter, not distinguished by gender. After weaning, fecal samples from each pup were individually collected. About 2–3 droppings, collected directly from the anus of each animal, were preserved in cryovials on an ice bed then stored at − 20 °C until shipment on dry ice to the Icahn School of Medicine at Mount Sinai. Forceps used for collecting droppings were washed and cleaned using sterile water and 1% sodium bicarbonate between each sampling to avoid cross contamination.

### Bacterial 16S PCR and sequencing

Rat fecal DNA was extracted using the QIAamp PowerFecal DNA Kit (Qiagen, Valencia, CA) following the manufacturer’s instructions. Total DNA concentration was determined by Qubit 2.0 Fluorometer (Life technologies, Norwalk, CT). The phylogenetically informative V3–V4 region of 16S rRNA gene was amplified using universal primer 347F/803R [33, 34] with dual-barcoding approach previously described [35]. The integrity of the 16S PCR amplicons was verified by agarose gel electrophoresis. The resulting ~ 460-bp sized amplicons were pooled and then sequenced with the Illumina MiSeq 2 × 250 paired-end sequencing platform at OCS genome technology center of New York University Langone Medical Center.

### 16S data analysis

The sequencing data were merged and filtered to remove the merged reads with a length of < 400 bp or the quality score of < Q30 at more than 1% of bases. Sequentially, all filtered high quality reads were split by dual-barcode and trimmed of primer regions using a self-defined bash script to integrate several sequencing processing commands from fastx [36], QIIME [37, 38], and seqtk [39]. Duplicated measurements of four sample were processed and sequenced using different barcodes to test the sequencing reproducibility. Five blank samples were also sequenced and referenced to filter the possible environmental contamination during the sample procession. The split high-quality reads were further processed by QIIME 1.9.0 [37]. We used the command *pick_open_reference_otus.py* with the defaulted green_gene 97_otus reference sequences to cluster of > 97% similar sequencing reads as an OTU using uclust [40]. Representative sequences for each OTU were aligned using PyNAST and build the phylogenetic tree. Finally, the QIIME generated biom-formatted OTU table contains the taxonomic information and absolute counts for each identified taxon in each sample.

The diversity within each microbial community, so-called alpha-diversity, was calculated using the Shannon Index [41] as metric and represented the measure of the diversity at the family and genus level. The overall microbiome dissimilarities among all samples were accessed using the weighted UniFrac distance matrices [42]. Non-metric multiple dimensional scaling (NMDS) were used to visualize the dissimilarities. The permutational multivariate analysis of variance PERMANOVA test [43], with the maximum number of permutations = 999, was performed to assess the significance of the overall microbiome differences between groups by collection timepoints and treatment. The PERMANOVA procedure using the [Adonis] function of the *R* package *vegan* 2.0–5 [44] partitions the distance matrix among sources of variation, fits linear models to distance matrices and uses a permutation test with pseudo-*F* ratios to obtain the *p* values. Using the LEfSe method [45], we further selected the microbiome features significantly associated to time of collection and treatments at various taxonomic ranks.

## Results

No unexpected clinical signs or symptoms were observed in the experimental animals during the in vivo phase. In particular, no sign of changes in maternal behavior during lactation (nesting and nursing) were observed during the experiment. There was no clinical evidence of alterations in activity or behavior in pups. Body weight, water and feed consumption both in dams and pups were no different across the groups. Litter sizes were fully comparable among groups, with mean number of live pups: control group 13.6 (range 10–16); glyphosate group 13.3 (range 11–17); Roundup group 13.9 (range 11–16).

We extract the total DNAs from 433 SD rat fecal samples. Following the timeline illustrated in Figs. 1, 120 fecal samples were collected from 24 F0 dams in three treatment groups and at five time points (before mating, GD5, GD13, LD7 and LD14). From F1 pups, we collected 313 fecal samples, in which 13 at PND 7, 24 at PND 14, 108 each at PND 31 and PND 57, and 60 at PND 125. We observed that the fecal samples of pups at PND 7 and PND 14 showed significant low DNA yields (Additional file 1: Figure S1A). We further performed microbiome survey on 433 SD rat fecal samples, and 5 water blanks using bacterial 16S sequencing on Illumina MiSeq 2 × 250 pair-end platform. After merging and filtering by read length > 400 bp and the quality score > Q30 at more than 99% of bases, we obtained ~ 2 million high quality reads (the average number of reads = 4576 per sample with standard deviation = 6567). The number of reads were not significant different by exposure type (Additional file 1: Figure S1A). The taxa composition was grouped by age and the exposure types and summarized in Additional file 1: Figure S1B. We also provided the complete taxonomic OTU tables in Additional file 2.

**Fig. 2:**
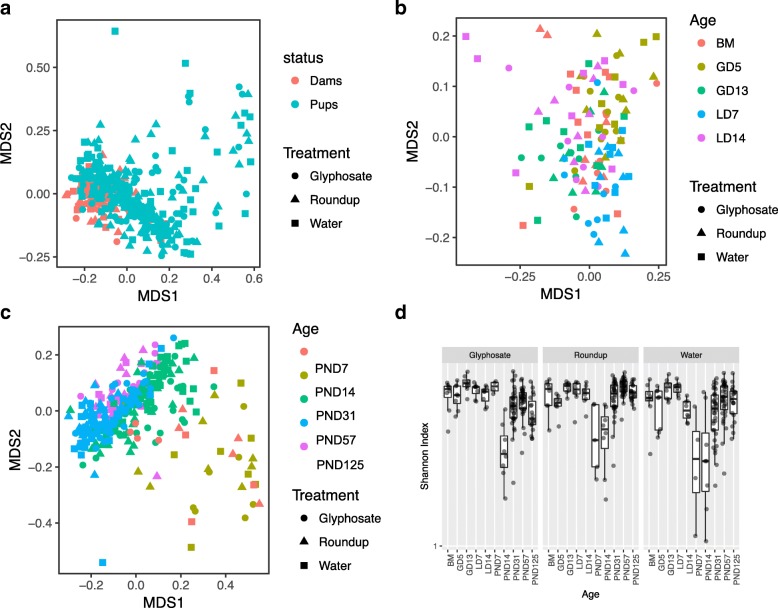
The overall microbiome diversity. **a**, **b**, and **c** are non-metric dimensional scaling (NMDS) plots visualize the overall microbiome dissimilarities (beta-diversity) between individual rat across time. **a** All samples from SD dams (pink) and pups (green) of three treatment groups; **b** All samples from SD dam rats only. Colors indicate sample collection timepoint. BM: before mating; GD 5: gestation day 5; GD 13: gestation day 13; LD 7: lactation day 7; and LD 14: lactation day 14. **c** All samples from SD pup rats only. Colors indicate sample collection timepoint. PND 7 to PND 125: postnatal day 7 to postnatal day 125. **d** Box plots show the mean and variance of the within-community diversity (alpha-diversity) measured by Shannon index in three treatment groups across all time of collections

The overall microbiome dissimilarity, defined by beta-diversity, was visualized by non-parametric multi-dimensional scaling (NMDS) plot of all samples (Fig. 2a), dams only (Fig. 2b) and pups only (Fig. 2c). We found that the early postnatal samples at PND 7 and PND 14 were far apart from the dams at LD 7 and LD 14 while the later postnatal samples at PND 31, PND 57 and PND 125 were clustering with the dams (Fig. 2a). The mean and variance of the within-community diversity (α diversity) measured by Shannon index showed that the samples from dams possessed higher, while early postnatal samples from pups showed lower α diversity (Fig. 2d). Student t-test showed significantly increased α diversity from PND 14 to PND 31 (*p*-value< 0.05 for all treatment groups) but no differences between samples at same age but different treatment group.

**Fig. 3:**
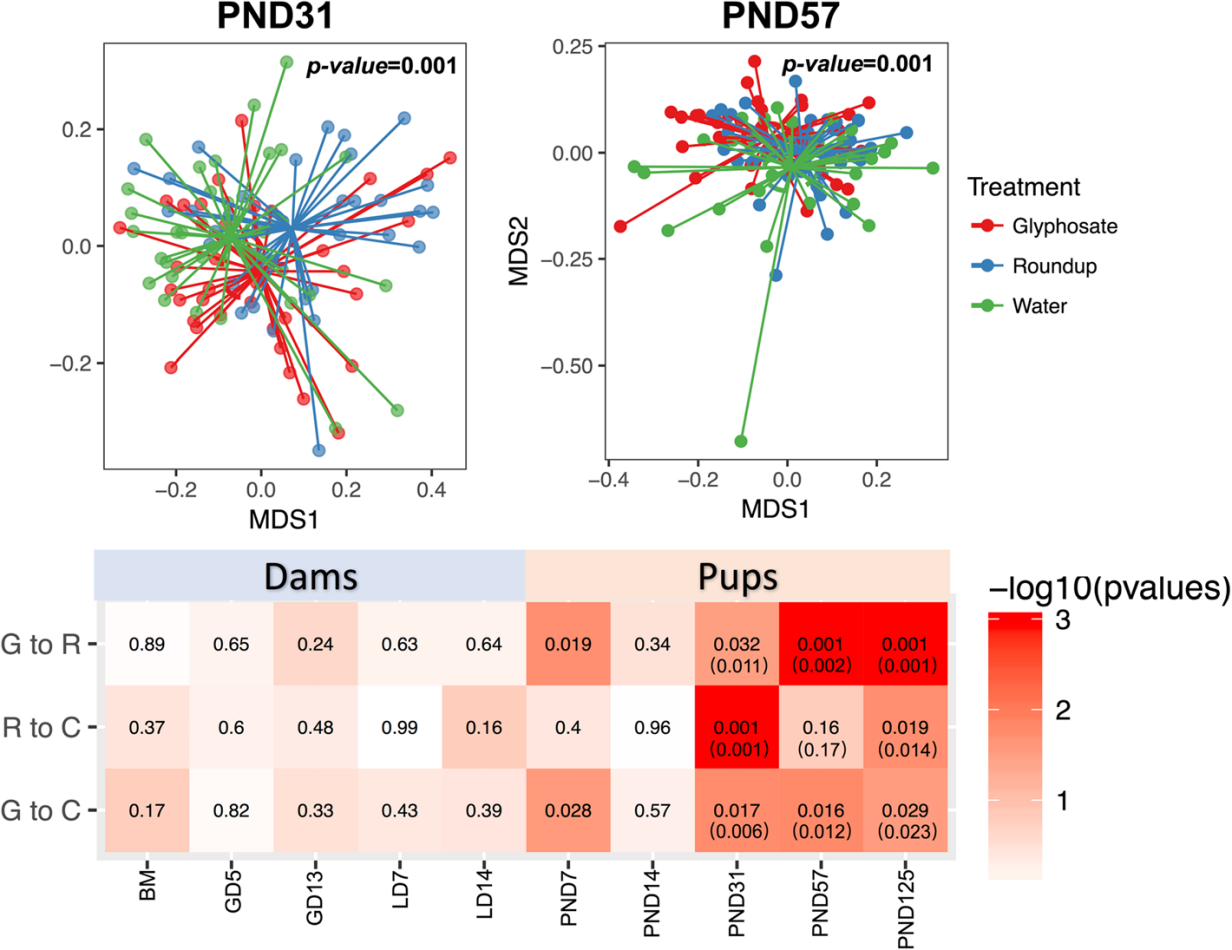
The effect of glyphosate exposure on overall microbiome diversity. **a** NMDS plots visualize the overall microbiome dissimilarities (beta-diversity) between individual rat of three treatments at PND 31 and PND 57. **b** PERMANOVA test is performed to test the significance among all three treatments (displayed in NMDS plots) and between two treatments (values are listed in tables). The *p*-values in parenthesis were adjusted for genders. G: glyphosate; R: Roundup; C: control water

We compared the overall microbiome changes by treatment at different age groups from pups and dams. Nonmetric multidimensional scaling (NMDS) plots visualized the overall microbiome dissimilarities by treatment at PND 31 and 57 (Fig. 3a). The PERMANOVA test was used at each age group to test the significance of the differences at overall rat gut microbiome between treatment and control. The test results (*p*-values shown in Fig. 3b) showed that the overall microbiome was significantly altered by both Roundup and glyphosate treatment compared to controls. Similarly, we also found significant differences in microbiota between Roundup and glyphosate exposed F1 pups. We also observed that the overall microbiome was significantly different by sex at PND 125 (*p*-value = 0.028, 0.007 and 0.013 by PERMANOVA test for Glyphosate, Roundup and control group, respectively). To adjust for the sex effect, we performed additional multivariable PERMANOVA test with both treatment and sex as predictive variables. We found that those test results were consistent (Fig. 3b). However, none of the F0 dam groups showed significant differences in overall microbiota diversity..

**Fig. 4:**
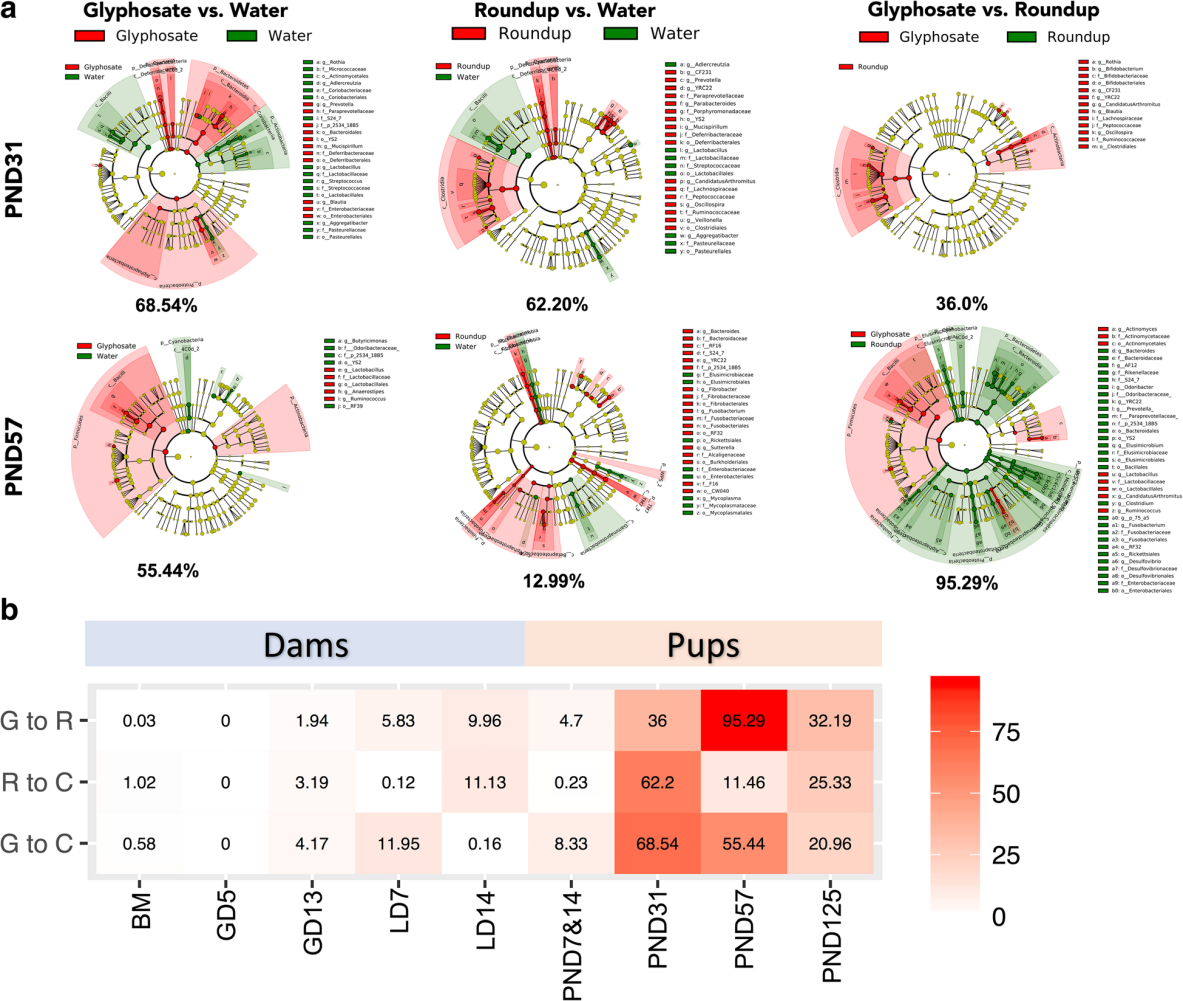
Differential microbial features selected via LEfSe between treatment. **a** Clad plots visualize the significant differential taxa features from phylum (inner circle) to genus (outer circle) at PND 31 and PND 57. Color indicates the more abundant taxa under each condition. **b** The table lists the overall abundance of the significant differential taxa between treatment across time

The linear discriminant analysis effect size (*LEfSe*) analysis was performed using 16S sequencing data from rat fecal samples in order to select particular discriminative features of the glyphosate exposure. Consistently with the overall microbiome changes by exposure at different age groups (Fig. 3), we found several significant differential taxa features associated with exposure. In particular, at PND 31, the results showed that the microbiota of both glyphosate and Roundup exposed pups had significantly higher prevalence of *Prevotella* genus (*Bacteroidetes* phylum) and *Mucispirillum* genus (*Deferribacteres* phylum) and lower prevalence of *Lactobacillus* genus (*Firmicutes* phylum) and *Aggregatibacter* genus (*Proteobacteria* phylum) (Fig. 4a 1–2). However, some of the selected features were treatment specific. For instance, among the most significant features with LDA score > 3.0 and p-value< 0.05, we found increased *Blautia* genus (*Firmicutes* phylum) and decreased *Streptococcus* genus (*Firmicutes* phylum) *and Rothia* genus (*Actinobacteria* phylum) only in glyphosate exposed PND 31 pups, but not in Roundup exposed samples. In contrast, increased *Parabacteroides* genus (*Bacteroidetes* phylum) *and Veillonella* genus (*Firmicutes* phylum) were only found in Roundup exposed pups, but not in glyphosate exposed samples at PND 31. Between two exposures (Fig. 4a 3), Roundup exposed pups showed increased *Clostridia* class (*Firmicutes* phylum), in particular, *Blautia* genus and *Actinobacteria* class (*Actinobacteria* phylum), in particular, *Rothia* and *Bifidobacterium* genera at PND 31. Furthermore, we found the treatment associated taxa features were not consistent at different postnatal time points. Many features selected at PND 31 did not appeared at PND 57 (Fig. 4a 4–6, Additional file 3: Figure S2), suggesting the less stability of early-life microbiota and continuous effect on gut microbiota by the exposure. When counting the total abundance % of the significant differential taxa by treatments, the pups showed much higher impact by exposure than the dams (Fig. 4b).

## Discussion

GBHs are the most applied herbicides worldwide; humans are commonly exposed to these environmental chemicals at a wide range of doses depending upon the job setting (farming vs. food consumption) and route of exposure (ingestion vs. inhalation). Environmental contamination from GBHs is now ubiquitous and residues of glyphosate has been found in air [46], groundwater [47], drinking-water [48], crops [49], food [50] and animal feed [51]. The possible effects of GBHs on human health are the subject of an intense public debate, for both its potential carcinogenic and non-carcinogenic effects, including endocrine disruption [52, 53], neurotoxicity [54], developmental and reproductive toxicity [55], autoimmunity [56], gastrointestinal disorders [57], obesity, diabetes [58–60], and other metabolic and cardiovascular disorders [61] and central nervous system dysfunctions such as learning and memory impairment, anxiety, stress, depression [62] and autism [63]. These chronic pathologies (non-communicable diseases – NCDs) may occur even at doses that are much lower than the ones considered during risk assessment, in particular during sensitive periods of life (such as fetal development) [7, 22].

Recent advances in human microbiome research suggested that the gut microbiome is a key player in human metabolism [64–66]. It is thus reasonable to hypothesize that exposure to environmental chemicals may modify the gut microbiome and its metabolites and ultimately influence human health. Microbiota-generated metabolites and their cellular and molecular components are increasingly being recognized as an essential part of human physiology, with profound effects on the homeostasis of the host organism. Unfortunately, determining the concentrations of these biologically active substances in target cells presents serious difficulties related to the extraction and processing of samples, especially faecal material, and the limitations of currently available measurement techniques [15]. Meta-omics and evolving computational frameworks will hopefully lead to the systematic prediction and discovery of more microbial metabolites and components involved in neuroendocrine, immune, metabolic, and epigenetic pathways.

Rats are proposed to be more representative of the human gut microbiota than mice because the gut bacterial communities of humanized rats more closely reflect the gut microbiota of human donors [67, 68]. We have previously used our animal model, SD rats, to study the effect of postnatal low-dose exposure to environmental chemicals on windows of susceptibility and on the gut microbiome. The study [69] showed the low-level phthalate, paraben and triclosan exposure altered the gut microbiome of adolescent rats. These results are consistent with other studies, indicating our animal model as a suitable model for studying microbiome [70, 71].

Since glyphosate has shown enzyme inhibition activity in plants and microorganisms, we therefore postulate that low-dose exposure to glyphosate or GBHs may also modulate the composition of the gut microbiome. In this study, when compared to the adult rat dams, the gut microbiome of pups at PND 7 and 14 showed lower taxonomical richness but higher variance within sample and higher sample-to-sample dissimilarity [69]. One pitfall of our study was that direct measurements of exposure to GBHs in milk was not performed [72]. In our pilot study we simply reproduced the human exposure, which includes lactation as only source of nourishment for pups from birth until around PND 21. The shortcomings concerning the analysis of glyphosate in breast milk are mainly related to the difficulty and stressing technical procedure for collecting milk from dams and to the complex nature of the breast milk matrix. Indeed, milk is an aqueous mixture of carbohydrates, proteins and fat. Analytical methods developed for watery matrices cannot be directly transferred to breast milk. In April 2014, a non-peer-reviewed report was published, in which glyphosate in breast milk of American mothers was detected in 3 out of 10 samples ranging from 76 to 166 ng/mL. In this study, the concentration of glyphosate in milk samples was determined by enzyme-linked immunosorbent assay (ELISA) [73]. The limit of quantification (LOQ) of the assay was given as 75 μg/L in milk. Other studies, based on liquid chromatography–tandem mass spectrometry (LC-MS/MS) and a gas chromatography–tandem mass spectrometry (GC-MS/MS) methods, have found no evidence of transfer of glyphosate into milk. Both methods have been fully validated and reported as suitable for the determination of glyphosate with an LOQ of 1 ng/mL [72, 74]. Nevertheless, future independent research is needed, considering different educational and ethnic backgrounds, location of residence (e.g., urban compared with rural), occupational and dietary glyphosate exposure and adequate sample size of the cohort.

Our results revealed that both glyphosate and glyphosate formulated Roundup, at doses admitted in humans, including children and pregnant women, significantly altered the microbiota diversity and resulted in prominent changes at multiple taxon in exposed pups. However, those effects on microbiota were not significant in the adult dams. Previous evidence has shown that the gut microbiota at postnatal age is less stable than at adult age and it changes over the first several years of life [75]. The maturation of the gut microbiota has been proven to be affected by multiple factors, for instance, diet, medications, host genetics, etc. [76]. Disruption of the microbiota during its maturation by low doses of various environmental chemicals has been showed to alter host phenotypes, such as weight, metabolism and other disease risk [77]. Our data suggests that the prepubertal age microbiota is more sensitive to GBH exposure compared to the adult microbiota, therefore the postnatal age is likely a “window of susceptibility” for GBHs to modulate the gut microbiome.

Furthermore, our results showed that the overall microbiome diversity and composition were significantly different between Roundup and glyphosate, suggesting possible synergistic effects of the mixed formulation on gut microbiota. As most of GBHs contains multiple surfactants and adjuvants might act differently than glyphosate alone, it is not only important to understand the individual effects of glyphosate, but also the synergistic impact of mixed formulations. In fact adjuvants might act alone or in a synergistic manner and increase the toxic effects of glyphosate [78–81].

In addition, both clinical and experimental studies showed impact of gut microbiota on the gut-brain axis (which mainly includes the immune, neuroendocrine, and neural pathways) [82–84] in an age-dependent manner [85]. Gut bacteria communicating with the host through the microbiota-gut-brain axis could influence brain and behavior [86]. In particular, the changes at postnatal microbiota may affect the neurvous system, reflecting by changes in levels of pituitary hormones including ACTH [83, 87], cortisol, BNDF [88] and etc. Sprague-Dawley rats represent an excellent animal model to explore these early-life effects as their microbiome is more similar to that of humans than the microbiota profile of mice [67].

This study has some limitations. First, the actual levels of GBHs that reached the fetus during gestation or through milk consumption postnatally by the offspring cannot be accurately estimated. Second, we only collected maternal feces so that we cannot fully evaluate the role of maternal microbiota in the fetal development without the maternal sample/data collection from oral, vaginal and other body sites. Indeed, in recent years it is becoming apparent that, besides breast milk, other sources could allow maternal-offspring microbial transfer. Rodents “inherit” their microbiomes in a similar fashion to all placental mammals, including humans: through vaginal delivery and close maternal association throughout the neonatal period (vertical transmission). Maternal vaginal, skin, mammary fecal and oral microbiomes, microbial spreading in bedding are efficiently transmitted to offspring and represent other possible mechanisms of maternal influences on pups intestinal colonization [89]. Finally, the microbiome survey used a cost-effective 16S amplicon targeted sequencing approach. This technique allows us to identify differential taxa compositions by exposure only to genus level. Additional meta-genomics and meta-transcriptomic analysis may need to visualize the functional and metabolic alternations and identify bacterial features at species/strain level. In addition, given the differences in taxonomic composition of the microbiomes of rats and humans, the extent to which the results of this analysis can be relevant to humans is not clear. Future work should investigate how the route and concentration of exposure impact the rat microbiome, and quantify how these perturbations may impact subsequent health outcomes. Nevertheless, these data strongly indicate that GBHs exposure can exerts biological effects early in development which may have long-lasting health effects later in life.

## Conclusion

Our pilot study provides initial evidence that maternal exposure to commonly used GBHs, at doses currently considered as acceptable in humans, is capable of modifying the gut microbiota in rat pups, in particular before puberty (PND 31). Further long-term investigations are necessary to elucidate if the shift in the microbiota induced by GBHs exposure is contributing to the downstream other health effects. Nevertheless, understanding the microbiota changes during this critical window of susceptibility could be of great importance for disease prevention. The potential health effects of GBHs during development, such as childhood, warrant further investigation.

## Additional files

Additional file 1: **Figure S1.** 16S microbiome profiling. **A**. Dot plot shows the distribution of the number of reads in three treatment groups. The Wilcoxon test significance between two groups was listed in table on the right and the diagonal of the table shows the average reads of each group. **B**. Box plot shows the mean and variation of total DNA concentrations from rat fecal samples. **C**. Bar plot showed the mean abundance of microbial composition at phylum level for each treatment and time of collection. (PDF 174 kb)

Additional file 2: 16S OTU table in biom format. (BIOM 6871 kb)

Additional file 3: **Figure S2.** The changes of *lactobacillus* and *Prevotella* during the time of sampling. Line plots show the mean and standard error of relative abundance% of *Lactobacillus* (upper figure) and *Prevotella* (lower figure) during the time of sampling from PND 7 to PND 125. (PDF 543 kb)

## Abbreviations

ACTH: Adrenocorticotropic hormone
AMPA: Aminomethylphosphonic acid
BDNF: brain-derived neurotrophic factor
CMCRC: Cesare Maltoni Cancer Research Center
DOHaD: Developmental origins of health and disease
ELISA: Enzyme-linked immunosorbent assay
EPSPS: 5-Enolpyruvylshikimate-3-phosphate synthase
EU: European Union
GBH: Glyphosate-based herbicides
GC-MS/MS: gas chromatography–tandem mass spectrometry
GD: Gestational day
GM: Genetically modified
LC-MS/MS: Chromatography–tandem mass spectrometry
LD: Lactating day
LDA: Linear discriminant analysis
LEfSe: Linear discriminant analysis effect size
LOQ: Limit of quantification
NCDs: Non-communicable diseases
NMDS: Non-metric multiple dimensional scaling
OUT: operational taxonomic unit
PND: Post natal day
PyNAST: Python nearest alignment space termination
QIIME: Quantitative insights into microbial ecology
RI: Ramazzini Institute
SD: Sprague-Dawley
US ADI: United States Acceptable Daily Intake
