# Supplementary figures and images for: The Ramazzini Institute 13-week pilot study on glyphosate and Roundup administered at human-equivalent dose to Sprague Dawley rats: effects on the microbiome
Source: Environ Health. 2018 May 29;17:50. doi: 10.1186/s12940-018-0394-x (PMC5972442; doi:10.1186/s12940-018-0394-x)

**A**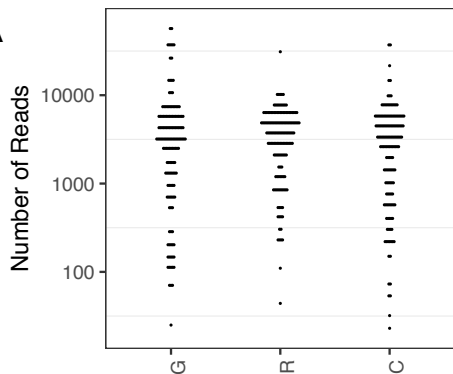

|   | G    | R        | C        |
|---|------|----------|----------|
| G | 5845 | $p=0.37$ | $p=0.47$ |
| R |      | 4054     | $p=0.07$ |
| C |      |          | 3995     |

**B**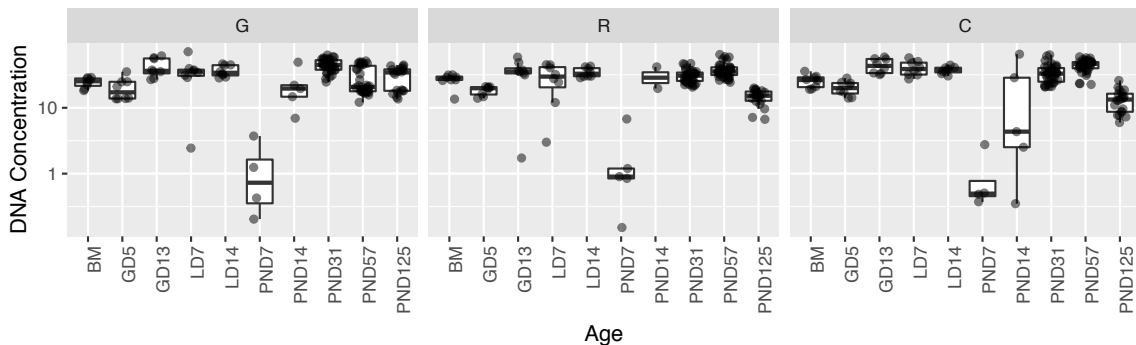**C**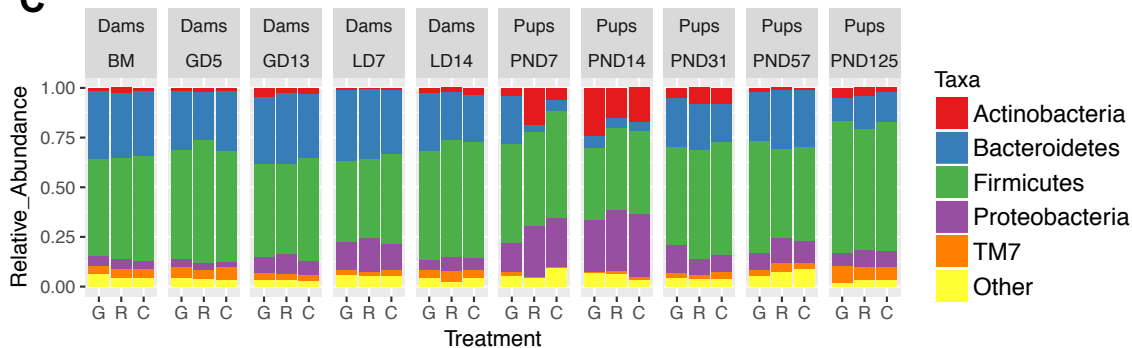

Supplement: Supplementary file 1 — Figure S1. 16S microbiome profiling. A. Dot plot shows the distribution of the number of reads in three treatment groups. The Wilcoxon test significance between two groups was listed in table on the right and the diagonal of the table shows the average reads of each group. B. Box plot shows the mean and variation of total DNA concentrations from rat fecal samples. C. Bar plot showed the mean abundance of microbial composition at phylum level for each treatment and time of collection. (PDF 174 kb) [file 12940_2018_394_MOESM1_ESM.pdf]

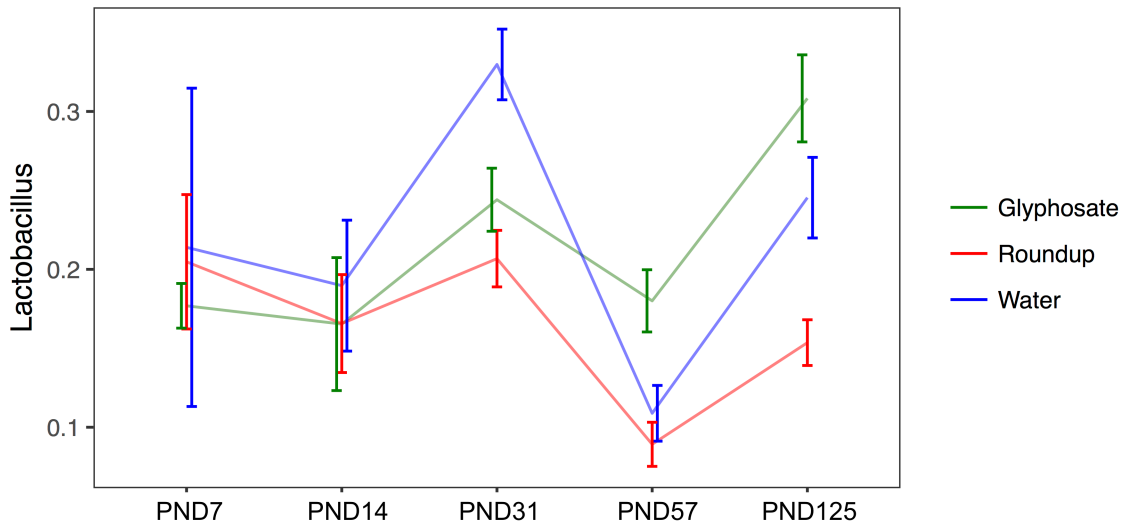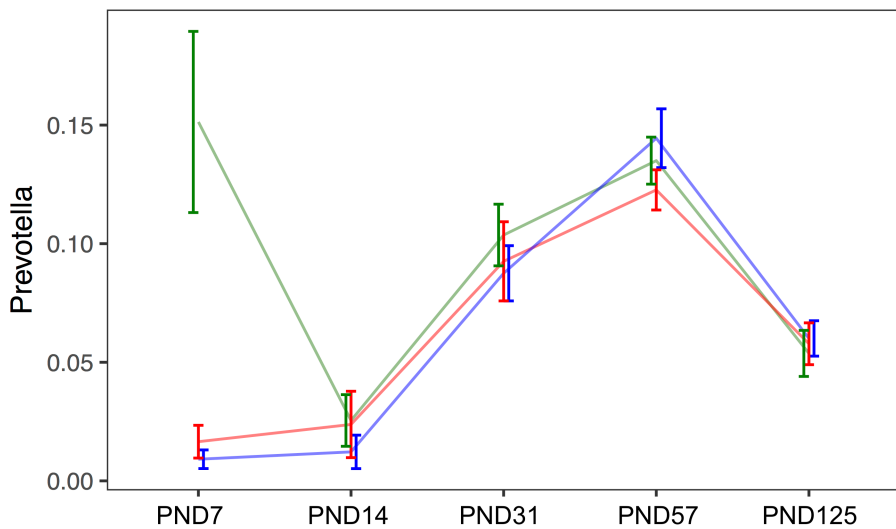

Supplement: Supplementary file 3 — Figure S2. The changes of lactobacillus and Prevotella during the time of sampling. Line plots show the mean and standard error of relative abundance% of Lactobacillus (upper figure) and Prevotella (lower figure) during the time of sampling from PND 7 to PND 125. (PDF 543 kb) [file 12940_2018_394_MOESM3_ESM.pdf]
